# The lure of siren’s song: Exploring the influence of destination song perception on potential tourists’ travel intentions

**DOI:** 10.1371/journal.pone.0283615

**Published:** 2023-08-16

**Authors:** Long Wei, Ting Shao, Xinwei Shi, Keqin Ren, Ying Qian

**Affiliations:** 1 Fashion Institute, Shannxi Fashion Engineering University, Xi ’an City, Shaanxi Province, China; 2 Institute of Tourism, Xinjiang University, Urumqi City, Xinjiang Uygur Autonomous Region, China; 3 Institute of Education, Bangkokthonburi University, Bangkok, Thailand; 4 Hebi Vocational College of Energy and Chemistry, Academic Affairs Office, Hebi City, Henan Province, China; The Hong Kong Polytechnic University, HONG KONG

## Abstract

Tourism destinations are the important research objects of tourism geography. And destination songs, as a media of tourism destination image, play a very important role in it. Based on the SOR model, this study constructs a theoretical relationship between destination song perception and tourism intention. A total of 317 valid questionnaires were collected from potential tourists through the Internet and SPSS v.26.0 and AMOS v.24.0 were used for data processing to verify these theories. The study found that destination song perception has a positive effect on travel intention, emotion induced, and destination image perception; Emotion induced has a positive effect on destination image perception and travel intention; Destination image perception has a positive effect on travel intention. Emotion induced and destination image play a mediating role individually and play a chain mediating role together in the effect of destination song perception on travel intention, and there is no significant difference between different mediating effects. This study attempts to explain the influence of destination songs on the travel intentions of potential tourists, and might be used for tourism destination marketing, especially in creation, promotion and management of destination songs.

## Introduction

Under the background of Covid-19 virus outbreak, as an important propaganda tool of the destination image, destination songs have penetrated public life. High valued and function-well songs can not only relate to the destination with the emotional, cognitive and behavior of tourists, but also rely on the information and communications technology to break the limit of time and space. Such as the Internet, it has become one of the most ideal publicity channels for the pandemic. In the tourism industry, the market often dominates the development of marketing. With the change of market demand, tourism marketing has experienced a process from channel to brand and then to product. In the product era, with the updating and iteration of marketing methods, sensory marketing has emerged from traditional marketing with its new ideas and sensory marketing theory has also become one of the most rapidly developing theories in the field of marketing in recent years. But after reviewing related studies, we found that there are more discussions related to visual marketing in this field, such as color [1–3], visual design [4, 5], package design [6], the research objects include video [7, 8], images [9]. The difference is that auditory marketing research mainly focuses on the speaking style of announcers [10], while auditory marketing communication media similar to destination songs are rarely discussed [11]. As one of the five marketing tools in sensory marketing, auditory marketing is an indispensable support for sensory marketing [12]. As the carrier of auditory marketing, sound plays a decisive role in the success of auditory marketing. According to the regularity of sound production, sound is divided into two types: music and noise, and music is based on the system of pleasurable music. Songs, especially destination songs, as a kind of music, are an excellent vehicle for aural marketing.

Similarly, in tourism geography, destination has been the most popular research object, for example tourism slogans, destination songs have always been shouldering the role of promoting the image of destinations. How to successfully build and promote destination image has always been a hot topic of discussion. Destination song is a valuable tool in many destination image promotions. It is not only the carrier of destination marketing, but also the main way for tourism enterprises to improve their brand image. Destination song is a key medium to auditory marketing and destination image communication. Although destination song has great development potential, its practical application is only at the level of traditional advertising, and its development is extremely limited. With the development of the Internet, the quantity of music elements surge, especially destination promotional songs. The emotion, destination image and behavioral intention of tourism consumers may be directly affected by destination songs. However, the current development of destination songs is slow, obviously an unbalanced situation occurred, which needs to be solved urgently.

Among the tourism groups, potential tourists are most likely to make tourism consumption. The emotion evoked by songs and the perception of destination image are particularly important, which are two key factors affecting the willingness to travel. Therefore, this paper selects potential tourists who have ever listened to the destination songs as the research object, and builds a theoretical model of the influence of destination songs perception on travel intention based on the SOR model, which has four dimensions, destination song perception, emotion induced, destination image perception and travel intention respectively. SPSS v.26.0 and AMOS v.24.0 be used in the study for the structural equation modeling empirical test and compute the results of the influence of destination song perception on travel intention.

The contribution of this study explores the influence of destination songs on tourists’ emotions and travel intentions, and preliminary explores the process of destination image construction in tourists’ minds. The theory of the influence of destination songs on travel intention is constructed and verified, which may promote the development of tourism marketing from the perspective of destination songs.

## Research review

### Research background

Music is a temporal and spatial art that can directly influence all aspects of a person’s feelings, emotions, thoughts, and behaviors [13]. Among these influencing factors, behavior is the final outward activity that is governed by thoughts and is the result of the transformation from the internal to the external of the individual, so the study of behavior and even consumption behavior is the most meaningful thing. In previous studies, scholars have demonstrated that different factors of music influence consumer behavior to different degrees, and these musical factors can be broadly classified into two categories: internal factors come from the music itself, such as the sound of a lower pitch or music that causes consumers to infer that the size of the product will be larger, while this gap narrows under visual influence only [14], the soundtrack of a large volume promotes consumers to choose more unhealthy foods while a small volume promotes consumers to choose more healthy foods [15], the timbre of violin, long piano and piano makes the promotion easier [16], music tempo affects perceptions [17], fast music increases the number of consumption in high density environments [18]. External factors come from components such as consumers’ perception, awareness, and evaluation of music, for example, when consumers taste beer bills they are influenced by different music that induces in their different emotions [19], adding sound to the interface of a social platform decreases users’ platform and thus affects brand trust [20], that liking music affects consumer perceptions [17], etc.

Similar studies are mostly focused in the field of marketing, although in the tourism industry, some scholars have explored the influence of music on tourists’ reactions in hotels [21], and some scholars even tried to discuss the influence of destination-related pop songs on tourists’ intentions to visit destinations different perspectives [22]. However, the influence of destination songs on tourists’ behavior, especially their travel intention, is still a very important research topic which needs to be further discussed from different perspectives. From the perspective of tourists, there are few research on tourism consumption behavior stimulated by destination songs. Tourism consumption, as a special consumption behavior, combines the attributes of general consumption behavior with the particularity of tourism itself. Starting from the essence of destination songs, it is valuable and necessary to understand the influence of destination songs on tourists’ travel intention to understand destination songs systematically and achieve the purpose of promoting tourism consumption.

Destination songs, a typical song related to destination, have attracted the attention of scholars early than 2020 and been studied as an important research object [22, 23]. In this study, destination songs can be used for destination promotion, and influence consumption intention and consumption behavior. Travel intention, refers to the desire of tourists to carry out travel behaviors, can be considered as the subjective probability and motivation of the tourists where they want to engage in travel and how much time and energy the tourists are willing to devote to [24]. Emotion induced, one of the most important results after listening to music, which is evoked by the destination songs. When tourists hear the destination songs, they will arouse the corresponding emotions, which is the process of emotion induced. Destination image perception is people’s perception of the destination, and is an important element of the destination brand. Destination brand not only refers to what images people have of the destinations but also displays what kind of relationship they have with it. [25], so that studying the tourists’ perception of the destination image can understand the destination brand.

### Theoretical framework and research hypothesis

In 1974 Mehrabian and Russell proposed the Stimulus-Organism-Response (SOR) model [26] to analyze the influence of environment on human behavior. The model consists of three components: stimulus (S), organism (O), and response (R). The stimulus (S) is the driving force that may influence the cognitive and emotional processes of consumers, and it includes all external environmental stimuli that consumers can touch, hear, see, and feel. The organismic variable (O) relates to the "processes and structures within the individual that lie between the stimulus and the final behavior or response", which consists of perceptual, psychological, sensory and reflective activities, specifically human emotions and cognition. The response variable (R) is the final behavior following the output outcome or consumer response, including psychological responses such as attitudes or behavioral responses [27]. The sensory marketing, which suggests that consumers’ perception, judgment and behavior can be obtained directly through sensory stimuli, which are mainly composed of five senses: sight, hearing, touch, taste and smell [12]. The SOR model gives a lot of room for sensory marketing. And sensory stimuli play an important role in influencing consumer choice and evaluation [28].

**Fig 1:**
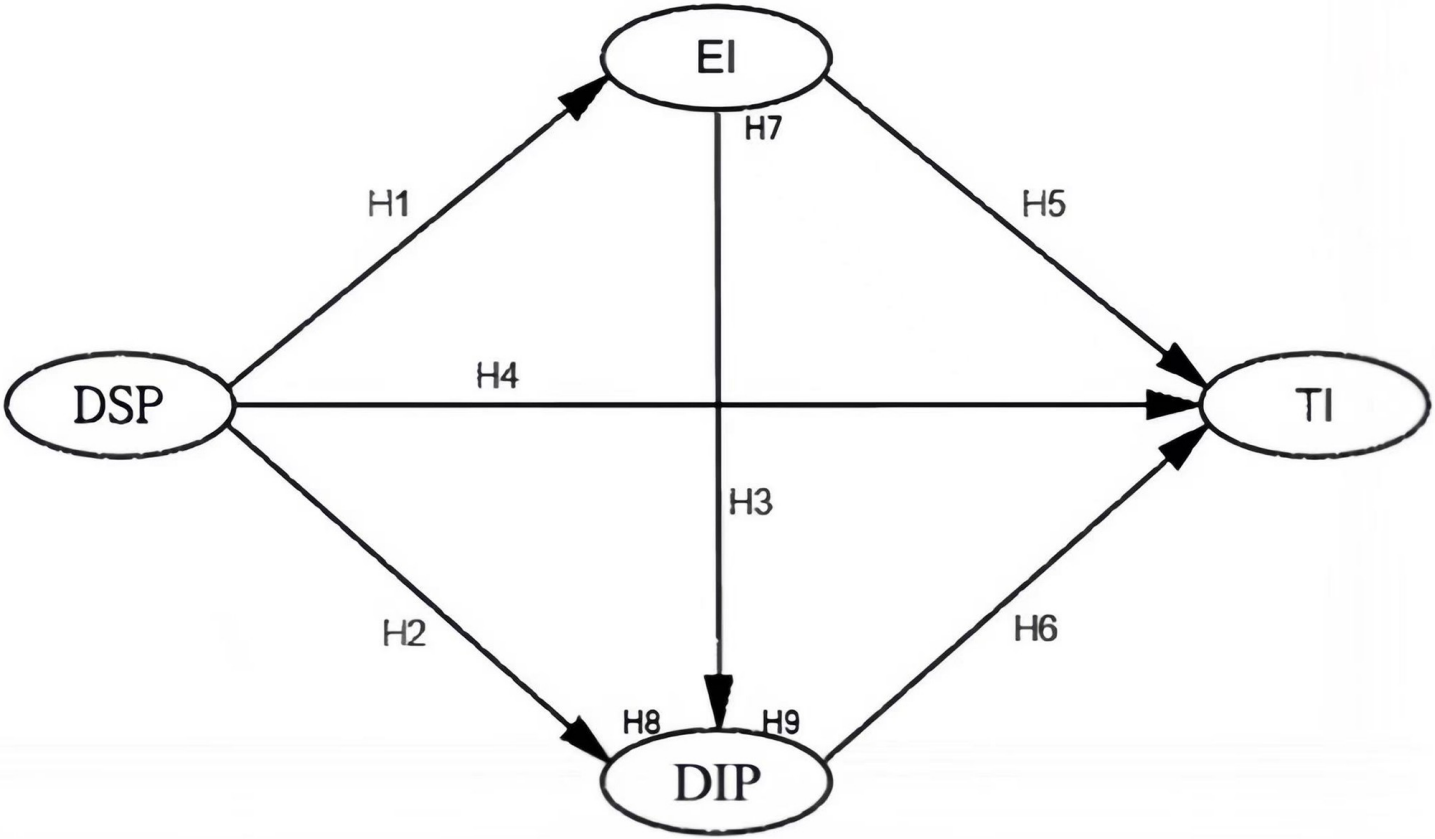
Research model.

In this paper, based on SOR theory and previous studies, hypotheses H1-H9 are proposed to establish the theoretical model of this study, as shown in Fig 1.

In contrast to other emotion-evoking techniques, the emotions evoked by music do not have any effect on the organismic or psychological integrity of the individual and are rarely related to personal adaptation and physical health. This type of emotion elicitation has a higher success rate [29]. In addition, specific emotions that are difficult to induce in other methods can also be relatively easily induced through music [30], and destination songs are relevant to the emotional evocation of potential tourists. Therefore, the following hypotheses are proposed in this study.

H1: Destination song perception (DSP) has a positive effect on emotion induced (EI).

Tourism destination image is the public’s overall and abstract perception and evaluation of a tourism place, and it is an intangible value that enhances the intrinsic and extrinsic spiritual values of the region, and a rational reproduction of the reality of the tourism place [31, 32]. Cognitive images, on the other hand, are images that are derived from facts. Due to the lack of prior experience of tourism products, the stimulus of external factors will form the sum of individuals’ beliefs and attributes about the object, which is the cognitive image. Jenkins argued that the image of tourist destination in tourists’ mind is influenced by individual factors and induced factors [33], and that tourists’ emotions induced by destination songs as one of the individual factors also affects tourists’ destination image. Thus, this study proposes the following hypotheses.

H2: Destination song perception (DSP) has a positive effect on destination image perception (DIP).
H3: Emotion Induced (EI) positively influences Destination Image Perception (DIP).

Travel intention is the subjective probability of a tourist to engage in a specific travel behavior, which is a subjective judgment of a person’s tendency to engage in a future travel behavior, and is the tourists’ intention to participate in a certain tourism activity [34]. And after the study by Reinoso-Carvalho et al. [19] it was found that there is some direct association between music and consumer behavior, which varies with the scenario. The intention to travel, as an antecedent intention to travel behavior, is also inevitably stimulated and influenced by destination songs as a form of music, and the potential tourists’ perception of destination songs will act as an external factor to stimulate the intention to travel and thus trigger the behavior of traveling. While Li S explored the effect of emotional responses triggered by destination TV commercials on the evaluation of tourism advertising effects in a study that verified the effect of emotions on travel intention [35], Hao X et al. in 2019 found that young viewers’ images of Iceland and the local tourism characteristics of the destination were consistent and related to their willingness to travel [36]. Based on the results of previous studies, it was inferred that the emotions, destination images and tourism intentions induced by destination songs were related to the following hypotheses.

H4: Destination song perception (DSP) positively affects tourism intention (TI).
H5: Emotion induced (EI) positively influences willingness to travel (TI).
H6: Destination image perception (DIP) positively influences tourism intention (TI).

Many tourist destinations use destination songs as an important vehicle for tourism marketing to attract tourists, and these songs can be found nearly everywhere. By listening to destination songs, potential tourists not only induce emotions, but also construct destination images, which can influence potential tourists’ travel intention. The relationship between potential tourists’ travel intention and the strength of emotion induced by these destination songs and the clarity of the induced destination image is corresponding, and it can be said that emotion and destination image play a certain mediating effect, and these mediating relationships are worth further investigation. Based on the above analysis, the following hypotheses are proposed.

H7: Emotion induced (EI) plays a mediating effect in the relationship between the influence of destination song perception (DSP) on travel intention (TI).
H8: Destination image perception (DIP) mediates the relationship between the influence of destination song perception (DSP) on travel intention (TI).
H9: Emotion induced (EI) and destination image perception (DIP) play a chain mediating effect in the relationship between the influence of destination song perception (DSP) on travel intention (TI).

## Sample and measurement

This study was approved by the Ethics Committee of Xinjiang University. We certify that the study was performed in accordance with the 1964 Declaration of Helsinki and later amendments. Written informed consent was obtained from all the participants prior to the enrollment of this study.

Research objects have already heard destination songs of potential tourists. Due to the limitation of the outbreak, this study adopts the method of convenient sampling, to ensure that the research object that potential tourists and visitors to distinguish with reality tourists. The research conducted on weekdays from November 1 to 20, 2021, through QQ group, WeChat group and online questionnaires to research object in the circle of friends. To ensure the authenticity and validity of the collected data, each account will be sent questionnaires only once. A total of 591 questionnaires were obtained, and 317 effective samples were finally obtained after screening questions, question-answering time and the normal distribution box diagram of the questionnaire. Effective samples were more than 20 times the number of questions in the maximum dimension of the questionnaire, it mets the requirement. The formal investigation analysis consists of reliability analysis and validity analysis (CFA). After reliability and validity analysis, AMOS V24.0 was used to conduct structural equation modeling path test and mediation effect test.

The questionnaire was composed of two parts. The first part contained filtering questions, “Have you listened to travel songs related to travel destinations in the last week?”, and demographic information, such as the gender, age, education background, monthly income, etc. In this part, respondents are required to fill based on their truth experience. To ensure the authenticity of the measurement results, respondents do not know the existence of filtering questions, so only the samples that answer "heard" in the filtering questions are selected as valid samples for data analysis. The second part measures the affect of destination songs, and these questions were divided into four dimensions: destination song perception, emotion inducted, destination image perception and travel intention. This part is based on the impression of potential tourists who have adopted a self-report report on it. Actually, the article measures content of songs and destination for potential tourists’ destination image perception. So, this paper there are 4 dimensions in the model. destination song perception, destination image perception, emotion induced and travel intention. All the scales selected in this study are mature scales that have been studied by predecessors and empirically tested. The questionnaire items are subject to Seven-point Likert scoring method. Table 1 for the source of the items.

**Table 1:** Scale development.

Dimension	Item	Source
Travel Song Perception	DSP1: I think the volume of the music is just right.	Min Z [23]
	DSP2: I feel the rhythm of the music is right.	
	DSP3: The style of music I feel is appropriate.	
Emotions Induced	EI1: You are full of emotion after listening to this song.	Zentner [37]
	EI2: You feel nostalgic after listening to this song	
	EI3: You are full of power after listening to this song	
	EI4: You are full of joy after listening to this song	
Destination Image Perception	DIP1: Overall, song-related destinations leave a good impression on me.	Prayag [38]
	DIP2: In general, I am very positive about the image of the destination associated with the song.	
Travel Intention	TI1: You may travel to a song destination in the next few years.	Xuefeng Ge [39]
	TI2: Next time you have a chance to travel, you will consider traveling to a destination related to songs.	
	TI3: You are very willing to travel to the destination of the song.	

## Result

This paper conducts descriptive statistical analysis on the demographic part of the 317 valid samples, in which there are slightly more women (62.1%) than men (37.9%); 37.9% are under 20 years old, 57.4% are between 21 and 35 years old, and 4.7% are older than 35 years old; in terms of education, 61.2% are undergraduates and 24.9% are masters or above, In terms of occupation, students accounted for 76.3%, teachers account for 3.5%, employees of enterprises account for 8.2%, self-employed persons account for 1.9%, employees of institutions account for 4.7%, and others account for 5.4%. The details are shown in Table 2, and no missing data are found in the sample.

**Table 2:** Respondent demographic characteristics.

		Frequency	Percent	Valid Percent	Cumulative Percent
gender	Men	120	37.9	37.9	37.9
	Women	197	62.1	62.1	100.0
	Total	317	100.0	100.0	
education	high school and below	9	2.8	2.8	2.8
	junior college	35	11.0	11.0	13.9
	undergraduate	194	61.2	61.2	75.1
	postgraduate and above	79	24.9	24.9	100.0
	Total	317	100.0	100.0	
profession	enterprise staff	26	8.2	8.2	8.2
	individual business	6	1.9	1.9	10.1
	student	242	76.3	76.3	86.4
	employees of government and public institutions	15	4.7	4.7	91.2
	teacher	11	3.5	3.5	94.6
	others	17	5.4	5.4	100.0
	Total	317	100.0	100.0	
income	less than 1500 RMB	213	67.2	67.2	67.2
	1500–3000 RMB	43	13.6	13.6	80.8
	3001–4500 RMB	23	7.3	7.3	88.0
	4501–6000 RMB	16	5.0	5.0	93.1
	6001–7500 RMB	9	2.8	2.8	95.9
	more than7500 RMB	13	4.1	4.1	100.0
	Total	317	100.0	100.0	
age	Under the 20	120	37.9	37.9	37.9
	21–35	182	57.4	57.4	95.3
	36–50	7	2.2	2.2	97.5
	More than 51	8	2.5	2.5	100
	Total	317	100	100	

Before performing statistical analysis on continuous variables, it is necessary to check the values of skewness and kurtosis of the data. If the absolute value of skewness of a variable is greater than 1 and the absolute value of kurtosis is greater than 7, then it implies that the variable is not normally distributed and there may be cases of outliers that need to be excluded from the sample. As shown in Table 3, the values of skewness and kurtosis in the sample are within the normal range and there are no outliers. The minimum and maximum values of the responses to the question items are significantly different, and the standard deviation shows the appropriate distance of each data deviation from the mean.

**Table 3:** Descriptive statistics.

	N	Min.	Max.	Mean	Std. Deviation	Skewness		Kurtosis	
						Statistic	Std. Err.	Statistic	Std. Err.
**DSP1**	317	1	7	5.823	1.105	-.989	.137	.581	.273
**DSP2**	317	3	7	5.953	.972	-.967	.137	.324	.273
**DSP3**	317	3	7	5.678	1.063	-.648	.137	-.530	.273
**EI1**	317	1	7	5.060	1.250	-.437	.137	-.229	.273
**EI2**	317	1	7	4.849	1.281	-.405	.137	.022	.273
**EI3**	317	1	7	4.820	1.269	-.247	.137	.084	.273
**EI4**	317	1	7	4.940	1.255	-.369	.137	.091	.273
**DIP1**	317	1	7	4.820	1.332	-.410	.137	.022	.273
**DIP2**	317	1	7	5.170	1.276	-.552	.137	.087	.273
**TI1**	317	1	7	5.268	1.233	-.757	.137	.479	.273
**TI2**	317	1	7	5.274	1.372	-.845	.137	.417	.273
**TI3**	317	1	7	5.186	1.373	-.730	.137	.192	.273

Reliability is one of the methods to test the stability of item data results. The higher the reliability, the higher the item reliable. SPSS V26.0 software is used to test the reliability of destination song perception, emotion inducted, destination image perception and travel intention. In the destination song perception (DSP) dimension, the Cronbach’s α coefficient value of item "DSP1" after deleting the item is 0.846. Slightly higher than this dimension, Cronbach’s α coefficient is 0.839. However, as the Cronbach’s α coefficient of destination song perception in the whole dimension is greater than 0.8, which is a rational range, and the increase is not obvious after deleting the item, it is considered to retain it. In the dimension of emotional inducted (EI), the Cronbach’s α (0.798–0.846) of each item after deletion is lower than the Cronbach’s α coefficient of the whole dimension (0.859), so these 4 items are considered to be retained. In the DIP dimension, Cronbach’s α coefficient of the overall dimension is 0.740, which is within the ideal range. Therefore, these two questions are retained. In the dimension of travel intention (TI), the Cronbach’s α value (0.784–0.814) of each item after deletion is lower than the Cronbach’s α coefficient of the whole dimension (0.855), so these three questions are considered to be retained. Table 4 lists the specific results.

**Table 4:** Reliability analysis table of total sample items (N = 317).

Items	The corrected term is related to the total	Cronbach’s α after deleting the item	Cronbach’s α	Total
**DSP1**	.635	.846	.839	3
**DSP2**	.770	.718		
**DSP3**	.712	.766		
**EI1**	.639	.846	.859	4
**EI2**	.680	.829		
**EI3**	.742	.803		
**EI4**	.754	.798		
**DIP1**	.588	-	.740	2
**DIP2**	.588	-		
**TI1**	.745	.784	.855	3
**TI2**	.730	.794		
**TI3**	.710	.814		

After checking the correlation between items in each dimension of the formal survey sample, it is found that the correlation between items in the four dimensions of destination song perception, emotion inducted destination image perception and travel intention were all greater than 0.3, the items in the survey questionnaire have good reliability.

The study used AMOS v.24 to conduct validation factor analysis (CFA) on the 317-sample data. The overall model has good fit, and the Factor Loading of the latent variable observations were greater than 0.6, indicating structural validity among the items, and their t-values are also significant at the 0.001 level, indicating that the scale has good convergent validity. The SMC is greater than 0.36, indicating that the reliability of the scale is in accordance with the standard, and the composite reliability (CR) is between 0.756 and 0.861, which are also greater than 0.7, indicating that the scale had high reliability. Average Variance Extracted (AVE) ranged from 0.610 to 0.665, which is greater than the criterion of 0.5, indicating that the scale has good discriminant validity (the results are shown in Table 5).

**Table 5:** Reliability and validity.

Dimension	Item	Factor Loading	Item Reliability	Composite Reliability	Average Variance Extracted
		Standardized	SMC	CR	AVE
DSP	DSP1	.697	.486	.848	.652
	DSP2	.881	.776		
	DSP3	.833	.694		
EI	EI1	.695	.483	.861	.610
	EI2	.720	.518		
	EI3	.836	.699		
	EI4	.860	.740		
DIP	DIP1	.663	.440	.756	.613
	DIP2	.886	.785		
TI	TI1	.847	.717	.856	.665
	TI2	.802	.643		
	TI3	.797	.635		

And the Pearson correlation between items is less than the square root of AVE, indicating that it had good discriminant validity (the results are shown in Table 6).

**Table 6:** Discriminant validity.

	**AVE**	**TI**	**DIP**	**EI**	**DSP**
**TI**	.665	**.815**			
**DIP**	.613	.620	**.783**		
**EI**	.610	.518	.611	**.781**	
**DSP**	.652	.471	.496	.457	**.807**

The diagonal in bold is the square root of AVE and the lower triangle is Pearson correlation.

The study use the AMOS v.24 to construct structural equation models from the 317 samples collected, and obtain the CMIN/DF value of 2.196 less than 3, which is within the ideal range [40]; the GFI is 0.948, the NFI is 0.936, IFI is 0.970, and CFI value is 0.970, all of which are greater than 0.9 in the ideal range [41–44]; RMSEA value is 0.062, which meets less than 0.08 in the ideal range [45]. Overall, the model fit is good.

Through the operation and measurement of AMOS, we verified nine hypotheses, and the test results are shown in Table 7. Destination song perception has a positive influence on travel intention (path coefficient 0.245**), emotional induction (path coefficient 0.515***) and destination image (path coefficient 0.314**), indicating that the higher the degree of destination song perception, The higher the emotional level induced by potential tourists, the better the destination image in mind, and the higher the desire to travel; Emotional induction has a positive influence on destination image (path coefficient 0.494***) and travel intention (path coefficient 0.213*), indicating that the higher the emotional induction level is, the better the destination image will be in the mind of potential tourists, and the higher the intention to travel to destination will be. Destination image has a positive influence on travel intention (path coefficient 0.499***), which indicates that the better destination image is in the mind of potential tourists, the more likely it is to inspire tourists’ intention to travel to the destination. Through path analysis, the direct effect (path coefficient 0.245***) and the three indirect effects (path coefficient 0.127***, 0.110*, 0.157*** *) are found to be valid, indicating that the mediation effect is valid, and they are all partial mediations. Although there are slight differences in the coefficients of the three paths, the differences are not significant after pair comparison. This shows that the songs not only directly affect the travel intention of potential tourists, but also affect the travel intention of potential tourists through emotional induction, destination image, emotional induction and destination image, and the importance of emotional induction, destination image, emotional induction and destination image are the same.

**Table 7:** Effect size of each path and results of the hypothesis test.

	Path	Point Estimate	Product of Coefficients		P	Bootstrapping		Inference
						Bias-corrected 95% CI		
			SE	Z		Lower	Upper	
**H1**:	DSP→EI	.515	.081	6.358	.000	-	-	supported
**H2**:	DSP→DIP	.314	.079	3.987	.008	-	-	supported
**H3**:	EI→DIP	.494	.085	5.791	.000	-	-	supported
**H5**:	EI→TI	.213	.094	2.267	.023	-	-	supported
**H6**:	DIP→TI	.499	.104	4.794	.000	-	-	supported
**Total Effect**	Total Effect	.639	-	-	.000	.473	.826	-
**Direct Effect**	Direct Effect (H4:DSP→TI)	.245	.091	2.687	.007	-	-	supported
**Indirect Effect**	Total Indirect Effect	.305	.066	4.621	.000	.193	.455	-
	Indirect Effect 1 (H9:DSP→EI→DIP→TI)	.127	-	-	.001	.058	.255	supported
	Indirect Effect 2 (H7:DSP→EI→TI)	.110	-	-	.056	-.003	.259	supported
	Indirect Effect 3 (H8:DSP→DIP→TI)	.157	-	-	.000	.066	.310	supported
**Difference Comparison between Indirect Effects**	Difference Comparison between Indirect Effect 1&2	.017	-	-	.859	-.155	.221	-
	Difference Comparison between Indirect Effect 2&3	-.047	-	-	.698	-.269	.160	-
	Difference Comparison between Indirect Effect 1&3	-.030	-	-	.605	-.154	.097	-

5000 bootstrap samples

## Discussion

The first important result from the analysis of the questionnaire data is that potential travelers are induced different emotions when they hear destination songs, the study counted the last time the respondents heard a destination song and found that 75.08% of the respondents listened to the song more than three days ago, which proves that destination songs induce emotions in listeners with a longer duration or that such induced emotions can be evoked according to specific conditions and ways. Evoked emotions can be evoked according to specific conditions and modalities with greater intensity and longer duration. As a second important result, the study found that destination songs and emotions play the role of "pencil" and "paint" respectively in the construction of destination images, and that tourists are influenced by destination songs to transform sound signals into visual signals, which outline the destination image. The tourist is influenced by the destination song to transform the sound signal into a visual signal, which outlines the overall image of the destination and makes it more three-dimensional through the coloring of emotions. This supports Jenkins 1999 that the image of the destination in the tourist’s mind is influenced by individual factors (emotions) and induced factors (destination songs). Regarding the results of primary concern of willingness to travel, the study found that when potential tourists hear destination songs that are induced with different emotions, the emotions positively influence them to construct an image of the destination, thus making them willing to travel; when potential tourists hear destination songs that construct an image of the destination, the image of the destination also makes them willing to travel; when potential tourists hear destination songs that also directly construct the intention to travel. This also supports the view of Li S 2018, Hao X 2019 and Reinoso-Carvalho 2019 et al [19]. The study also found that specific tourist intentions differed depending on the group of tourists; those under 35 years old were more likely to be influenced by destination songs and thus motivated to travel than those over 50 years old, and women tourists were more likely to be motivated by destination songs, emotions, and destination images than men tourists. The reasons for this phenomenon can be broadly attributed to the following: destination songs are mostly pop songs that fit the listening habits and aesthetics of young people, thus giving them a sense of closeness and making them more likely to be motivated to travel; young people are generally less socially experienced and more impulsive than older people; and the women group is more emotional than the men group.

To further confirm the specific factors in the destination songs that affect the willingness to travel, the study ranks the names of the destination songs collected in the questionnaire and selected the top songs to collect and analyze the song comments in NetEase Cloud Music, QQ Music and other platforms.

The study found that the direct influence of destination songs on the willingness to travel "I went to Chengdu after listening to Zhao Lei’s Chengdu; I went to Dali after listening to Hao Yun’s Dali; I went to Beijing after listening to Wang Feng’s Beijing Beijing; I went to Guangdong after listening to Guangdong Love Story by Guangdong Rain God. (Comment from the song Chengdu)" "The first time I heard this song, I thought I had to go to Dali, on my own. (Comment from the song Go to Dali) "There are many specific reasons, such as lyrics, melody, rhythm, arrangement, language, singing and other explicit factors of the song, and other invisible factors such as the story behind the song, cultural background, etc. "Listen to the song not only to listen to the arrangement, lyrics, and its background story. (Comment from the song The Shepherd of the Cocoanut Sea)" "The Xi’an language that gives people goose bumps is great! (Comment from the song Xi’an People’s Song)", these factors also influence the mediating variable of emotional triggering "listening to the song Shepherd of the Cocoanut Sea" and destination image perception "Indeed, Li Na sings well not only in the high notes, but also in the image of the destination. It is not only the soprano, but also sings the kind of vast and empty, thick, the kind of snowy plateau divinity. (Comment from the song Qinghai-Tibet Plateau)" "I listened carefully to each line of the lyrics, in addition to listening to it, I could also imagine the scenes he wrote with the lyrics, it really makes people very healing. (Comment from the song Chengdu)", and through these intermediary variables affect the willingness to travel "The first time I heard "Chengdu" is in the high school party, at that time did not know what song it is, just moved to the ground. The first time I heard "Chengdu" was at my high school party, and I didn’t know what it was about, but I was moved to tears by it. I finished my goal of going to school this September, and I’m going to Chengdu next year. (Comment from the song Chengdu)".

## Conclusion and prospect

### Conclusion

Based on the SOR theoretical model, this paper selected four variables: destination song perception, emotion elicitation, destination image and travel intention, than explored the relationship between destination song perception and potential tourists’ travel intention by constructing a structural equation model and analyzing song reviews. The following conclusions were drawn.

1. In the field of sensory marketing, the model of destination song perception on potential tourists’ travel intentions validates the SOR model and fill in the research gaps related to destination songs, which may explain the internal mechanism (especially in emotion and destination image) of the influence of destination songs on tourists to a certain extent.
2. Destination song perception not only significantly induces potential travelers’ emotions, but also stimulates potential travelers to construct destination images, which ultimately has a significant impact on travel intention. The higher the quality of the destination song, the higher the degree of emotional evocation and destination image construction, and thus the higher the willingness to travel.
3. The emotions of potential tourists induced by the destination song will stimulate the construction of the destination image and thus have an impact on travel intentions. The emotions induced by the destination songs of potential tourists as an individual factor awaken the subconscious destination image of tourists, while the construction of the subconscious destination image of potential tourists can only be obtained from other external channels or subjective perceptions, which are related to personal experiences. After receiving the stimulation of the information contained in the destination song, the potential tourist awakens the previously constructed destination image when the emotion is induced, thus affecting the travel intention.
4. Emotional evocation, destination image, and travel intention are influenced by the explicit and invisible factors of destination songs. The explicit factors of destination songs often include the visible factors of the song itself, such as lyrics, song, melody, arrangement, singer’s performance of the song. While the invisible factors of the song often include the story behind the song or the cultural background of the song. The explicit and invisible factors of the destination songs together determine the tourists’ judgment of the songs, and the judgment standard often varies with the songs and the tourists.

### Suggestion

In the future study of destination songs, this paper gives the following suggestions from the components of the destination song and its theoretical framework:

1. Destination songs contain lyrics, songs and other components. In future studies, different song elements can be subdivided and explored with different research methods.
2. At present, there are not much theoretical research related to destination songs. In addition to the three variables focused on this study, future managerial research can also focus on other variables related to tourists.

In the future practical application of destination songs, this paper puts forward the following suggestions on the creation, promotion and management of destination songs.

1. When creating destination songs, it is better to take into account the image of the tourism theme and the aesthetics of the main audience groups and adopt appropriate perspective with the background of culture or story, using the lyrics, melody, rhythm and other elements of the song to create the atmosphere and mood of the song. So that listeners can more easily induce emotions and resonate, and build the image of the destination more quickly, finally, it promotes tourism development. The choice of the song performer should be pondered will who has the ability to express the content of the song with a wide range of influence.
2. In terms of publicity, destination songs can be disseminated not only in the form of recordings, song websites and tourism social media platforms, but also in the form of tourism destinations, destination videos or movies, and tourism performances. Depending on the unique form of tourism, to expand the influence of the combination of push and pull.
3. In the process from production to promotion of destination songs, a complete plan should be made to integrate all resources to achieve the best quality and to avoid the risk of possible disputes including copyright and usage rights in advance. In the process of promotion, the responsible department should not only broaden the promotion path with the development of society, but also summarize the management experience in real time and make timely adjustment to the relevant management regulations so as to obtain the optimal management path.

### Limitation

1. Due to the limitations of epidemic situation and other factors, the samples of this study are selected in China, which may lead to cultural bias caused by cultural differences. In future studies, the further research will try to use different research methods to explore the results.
2. This study measures destination songs from the perspective of potential tourists’ perception of destination songs. The measurement perspective is relatively single. Future research can be carried out from different perspectives to expand the research results.
3. The questionnaire is self-reported by the subjects, and the measurement information related to emotions and other psychology may not be 100% restored to the real situation, which may cause some deviation of the results. Therefore, different measurement methods can be considered for future studies.
4. In addition to emotion and destination image perception, there may be other mediating variables in the research on the impact of potential tourists’ destination song perception on their intention to travel, which should be focused on in subsequent research.
